# TiO_2_/PDA Multilayer Nanocomposites with Exceptionally Sharp Large-Scale Interfaces and Nitrogen Doping Gradient

**DOI:** 10.1021/acsami.3c18935

**Published:** 2024-02-13

**Authors:** Jakub Szewczyk, Igor Iatsunskyi, Paweł Piotr Michałowski, Karol Załęski, Cassandre Lamboux, Syreina Sayegh, Elissa Makhoul, Andreu Cabot, Xingqi Chang, Mikhael Bechelany, Emerson Coy

**Affiliations:** † NanoBioMedical Centre, Adam Mickiewicz University, Wszechnicy Piastowskiej 3, 61-614 Poznan, Poland; ‡ Institut Européen des Membranes, IEM, UMR 5635, Univ Montpellier, CNRS, ENSCM Place Eugène Bataillon, 34095 Montpellier Cedex 5, France; § Łukasiewicz Research Network—Institute of Microelectronics and Photonics, Aleja Lotników 32/46, 02-668 Warsaw, Poland; ∥ Advanced Materials Department, Catalonia Institute for Energy Research (IREC), Sant Adrià de Besòs, 08930 Barcelona, Spain; ⊥ ICREA, Pg. Lluís Companys 23, 08010 Barcelona, Spain; # Gulf University for Science and Technology, GUST, 32093 Hawally, Kuwait

**Keywords:** atomic layer deposition, free-standing films, photocatalysis, bandgap, heterojunction

## Abstract

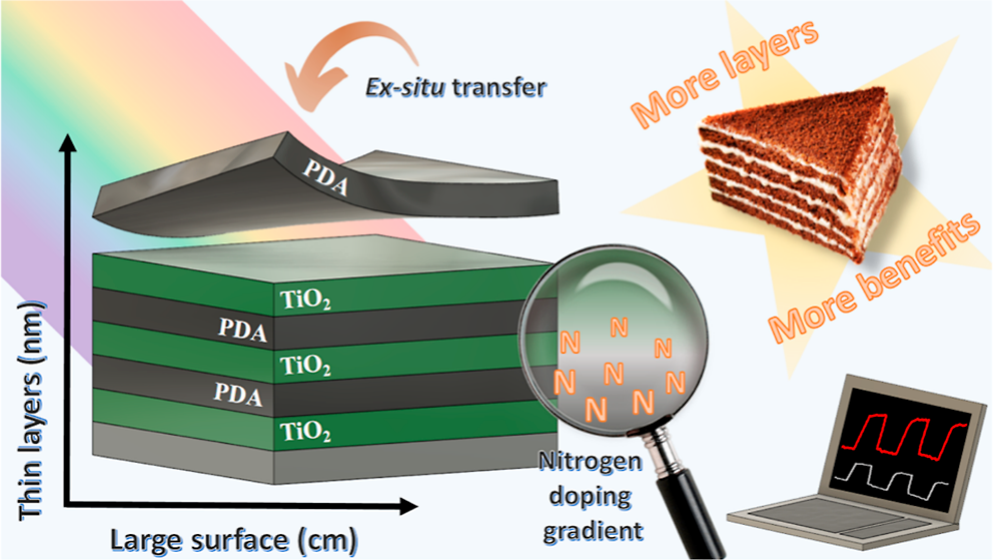

The evolving field of photocatalysis requires the development of new functional materials, particularly those suitable for large-scale commercial systems. One particularly promising approach is the creation of hybrid organic/inorganic materials. Despite being extensively studied, materials such as polydopamine (PDA) and titanium oxide continue to show significant promise for use in such applications. Nitrogen-doped titanium oxide and free-standing PDA films obtained at the air/water interface are particularly interesting. This study introduces a straightforward and reproducible approach for synthesizing a novel class of large-scale multilayer nanocomposites. The method involves the alternate layering of high-quality materials at the air/water interface combined with precise atomic layer deposition techniques, resulting in a gradient nitrogen doping of titanium oxide layers with exceptionally sharp oxide/polymer interfaces. The analysis confirmed the presence of nitrogen in the interstitial and substitutional sites of the TiO_2_ lattice while maintaining the 2D-like structure of the PDA films. These chemical and structural characteristics translate into a reduction of the band gap by over 0.63 eV and an increase in the photogenerated current by over 60% compared with pure amorphous TiO_2_. Furthermore, the nanocomposites demonstrate excellent stability during the 1 h continuous photocurrent generation test.

## 1 Introduction

Heterogeneous photocatalysis shows great potential in many fields of large interest, such as water remediation,^1^ air purification,^2^ and renewable energy conversion and storage, e.g., water splitting and solar fuel production.^3^ However, for heterogeneous photocatalysis to become widespread, cost-effective methods of production of high-performance photocatalytic materials at a commercial scale need to be developed.^4^ One particularly promising class of materials is hybrid organic/inorganic composites. In particular, oxide/polymer composites combine the advantages of both inorganic materials offering proper optoelectronic properties and polymers offering ample parameter tunability, flexibility, and stretchability.^5^ An additional advantage of polymers is that they can often be obtained from natural resources, i.e., biomass, or using environmentally friendly reagents. One of the most exciting polymers considered in the context of hybrid materials for photocatalysis is polydopamine (PDA), which was discovered from the inspiration of sea shells.^6^ PDA has catechol and amine functional groups, which enables it to adhere strongly to virtually any surface, and at the same time exhibits the properties of an organic semiconductor, which makes it a powerful surface modification material in band structure engineering and electron transfer processes.^7^ PDA has also an outstanding ability to generate photocatalytic heterojunctions with plasmonic,^8^ transition metal oxide,^9,10^ and sulfide^11,12^ nanoparticles. PDA might seem to be a supreme candidate for obtaining large-scale layered nanocomposites serving both as an adhesive interlayer^13^ and heterojunction-promoting functional coating.

We have previously shown that PDA free-standing films produced at the air/water interface (a/w-PDA) are characterized by a 2D-like laminar structure,^14^ which can be obtained on a large scale (up to several cm) while maintaining homogeneity and continuity,^15^ and are easily transferable to any desired substrate due to their extraordinary mechanical properties.^16^ Ex situ transferred PDA is a thin layer of PDA that is free-standing on the water surface and can be transferred to another desired surface. Such nanometrically thin a/w-PDA films transferred ex situ to the surfaces of semiconductors (ZnO and TiO_2_) can create efficient and stable heterojunctions useful in photocatalytic reactions.^17^

On the other hand, TiO_2_ is a promising photocatalyst that constitutes the basis for the construction of novel hybrid nanomaterials with high application potential,^18−20^ but simultaneously it suffers from poor efficiency due to a relatively large bandgap and fast charge recombination rate. TiO_2_ nitrogen doping is one useful modification to increase the efficiency of this inorganic semiconductor by reducing the bandgap, broadening the light response, and increasing the number of photogenerated carriers.^21,22^ However, there are still challenges associated with the formation and utilization of N-TiO_2_. First, the exact mechanism of band gap reduction by N doping of TiO_2_ is still unclear and needs further research. Also, control of the N doping concentration is essential and achieving successful optimization within various process conditions and nitrogen sources is challenging. Finally, the stability of N-TiO_2_ is still questionable; therefore, it is desirable to protect the N-TiO_2_ films against decomposition, e.g., passivation with a tight protection layer.^23^ The production method of N-TiO_2_ with superior photocatalytic activities utilizing simple facile techniques through the green routes is an emerging research topic.^24^

Atomic layer deposition (ALD) is one of the most commonly used titanium oxide (TiO_2_) deposition methods due to several advantages, such as precise thickness and composition control of the obtained conformal oxide layers—particularly important aspects in the production of advanced hybrid nanoarchitectures.^25^ Using mild temperatures (below 200 °C), an amorphous, high-quality thin TiO_2_ film can be obtained. Additionally, it can be deposited on virtually any selected substrate, e.g., polymer, as in this experiment—on PDA. In turn, during the thermal annealing of PDA, the reported product is nitrogen-doped graphene rather than graphite or graphene.^26,27^ This is because PDA, as a polymer with rich pyrrolic-N groups, serves as a nitrogen source. Profoundly, the positive correlation between the content of graphitic nitrogen, enhanced conductivity, facilitated electron transfer, and improved specific capacity was found.^27,28^ Whether PDA subjected to mild temperature treatment can be an efficient nitrogen source is unknown. However, previous reports on ALD processes on polymer substrates show that precursors can penetrate and react with them.^29,30^ Importantly, it was shown that the degree of this reaction can be increased by extending the precursor exposure cycle time in the ALD sequence.^29^ Predictions should be made that the growth of the layer with each ALD cycle will decrease the exposure of the polymer substrate, consequently leading to gradient-like phase formation.

Bearing in mind PDA’s ability to create a functional heterojunction on the surface of the inorganic semiconductor, we decided to arrange several layers alternately, thanks to which the number of interfaces obtained could be multiplied, thus the effect of heterojunction. The semiconductor chosen for modification was TiO_2_ due to several key features of the TiO_2_ nanofilms—a flat surface, excellent adhesion to diverse substrates’ surfaces, very well described band structure, and nontoxic behavior.^31^ However, another aspect was the most important, i.e., the possibility of obtaining a completely new type of heterogeneous organic/inorganic nanocomposite. We hypothesized that the ALD of TiO_2_ on the PDA layer may lead to the formation of a gradient interface, i.e., PDA/N-TiO_2_/TiO_2_. Additionally, PDA should act as a protective layer for unstable N-TiO_2_, and multiplying the number of layers will create a larger number of functional interfaces between the polymer and doped titanium oxide. Considering that amorphous TiO_2_ contains more defects and disorder than crystalline TiO_2,_ it is predicted to be more likely to accept nitrogen.^32^ Therefore, we used a low process temperature (200 °C) and an extended ALD cycle time. Ultimately, the conductive properties of PDA have not yet been well described, and it is not known whether it would ensure good electrical contact with the substrate.^33^ Therefore, the first layer of the multilayer composite should be TiO_2_, while the outermost layer should be PDA, which will increase the stability of the entire multilayer structure. In this way, we have developed a simple and reproducible path for obtaining a new type of multilayer composite, which we obtained on a large scale. Moreover, we present strong evidence for gradient-like nitrogen doping of TiO_2_ layers, which opens up many possibilities for constructing intelligent organic/inorganic interfaces.

## 2 Materials and Methods

### 2.1 Chemical Reagents

Materials in all synthesis procedures were used without any further purifications. Dopamine hydrochloride (CAS: 62-31-7, *s*, >98%), Trizma base (CAS: 77-86-1, *s*, >99%), hydrochloric acid (CAS: 7647-01-0, l, 25%), silicon wafer (Si 100, CAS: 7440-21-3, *s*), titanium tetrachloride (CAS: 7550-45-0), sodium sulfate (CAS: 7757-82-6, *s*), quartz (fused, thickness: 1.0 mm), and quartz/indium tin oxide (ITO) substrates (CAS: 50926-11-9) purchased from Sigma-Aldrich and ultrapure deionized water obtained from a Hydrolab Ultra UV system were used.

### 2.2 Synthesis of the PDA Free-Standing Films

The synthesis of PDA free-standing films was carried out in the specific conditions that we have determined in our previous work to achieve homogeneous and large-scale thin films with nanometer-scale control of the thickness and easy transfer onto the desired substrate.^15^ Dopamine in the form of dopamine hydrochloride was added to a Petri dish (8 cm in diameter and 2 cm in height) containing tris buffer solution (10 mM, 45 mL) to obtain a dopamine concentration equal to 0.5 mg mL^–1^. Stirring (300 rpm) took place on a magnetic plate throughout the synthesis time, and a glass lid covered the vessel with a small gap to allow oxygen flow and, thus, air exchange.

### 2.3 TiO_2_ Film Deposition

TiO_2_ layers were deposited by the ALD, which is described in detail elsewhere.^34−36^ Briefly, process conditions were as follows: temperature: 200 °C, purge gas: Argon, TiO_2_ precursors: H_2_O and TiCl_4_, number of cycles: 400, and process time: ∼12 h. The relatively long ALD time was related to longer breaks between cycles for the individual precursors. The aim was to keep the samples at a higher temperature and vacuum for longer so that the nitrogen diffusion process at the PDA/TiO_2_ interface could occur. To see the thickness profile of all the obtained layers, see the Results and Discussion section. Substrates were prepared to enable different characterization methods, such as bare silicon (100) wafers for the chemical and structural characterization, quartz glass for the UV–vis transmission spectroscopy, and quartz glass covered by ITO for the photo-electrochemical tests. All substrates had dimensions of no smaller than 1 × 1 cm.

### 2.4 Physicochemical Characterization

Raman Spectroscopy was performed using a Renishaw instrument equipped with microscope enclosure RE04, 633 nm laser source, and Leica objective lens ×50. The number of accumulations was 3. Exposure time was set to 0.1 s with 0.1% of the power of the laser source. X-ray diffraction (XRD) characterization was executed with the use of an MRD-X’pert^3^ diffractometer (PANalytical), operating at 45 kV and 40 mA with a Cu Kα radiation source (wavelength of 1.54 Å). The lamellae for high-resolution transmission electron microscopy (HRTEM) investigations were prepared by focused ion beam (FIB) JEOL, JIB-4000. The procedure is described in detail elsewhere.^37^ HRTEM was performed with a JEOL ARM 200F (200 kV). Secondary ion mass spectrometry (SIMS) measurements were performed with the CAMECA IMS SC Ultra instrument. To ensure sub-nanometer depth resolution, cesium with ultralow impact energy (100 eV) was used as primary ions, and the polarity of the detector was negative.^38^ X-ray photoelectron spectroscopy (XPS) was performed using KRATOS/AXIS Ultra DLD, X-ray source: Al Kα, 1486.6 eV; fwhm resolution 0.45 eV; acquisition time 0.1 s. Stationary transmission UV–vis spectroscopy was applied to investigate the bandgap, using deuterium–halogen light source AvaLight-DHc (Avantes), AvaSpec-Mini2048CL spectrometer (Avantes) and an optical fiber capable of operating in the broad UV–vis spectrum (220–800 nm). Obtained spectra of TiO_2_ (an indirect band gap semiconductor) were transformed^39^ and plotted against the photon energy. To obtain the optical constants of PDA and TiO_2_ films, spectroscopic ellipsometry analysis was performed using the SENTECH GmbH SER800 ellipsometer at incidence angles of θ = 60, 65, and 70°. This analysis covered the spectral range from 400 to 1000 nm with a scanning interval of 1 nm. In order to extract the refractive index and extinction coefficients of the layers, we employed the Bruggeman effective medium approximation. The TiO_2_ layers in the nanolaminates were described by using the Tauc–Lorentz dispersion function, while the PDA layers were characterized by using an advanced combination layer dispersion, consisting of Tauc–Lorentz and Brendel oscillator dispersions. During the regression analysis, the optical constants of the nanolaminates were determined, with the initial thicknesses of the layers being fixed.

### 2.5 Photo-electrochemical Experiments

Photo-electrochemical studies were carried out using a Gamry Reference 620 potentiostat in a three-electrode system where the investigated sample was a working electrode, Pt-mesh was a counter electrode, and Ag/AgCl/0.1 M KCl was a reference electrode in 0.5 M Na_2_SO_4_ electrolyte solution. Linear sweep voltammetry (LSV) experiments were carried out in a potential range from 0.5 to +1 V where the linear increase of potential was equal to 10 mV/s, and the light/dark cycle length was 1 s. Chronoamperometry (CA) experiments were developed by switching potential in a step-manner from 0 to 1 V and later keeping the system at a target voltage of 1 V for stabilization during 100 and 3600 s. Open circuit photopotential (OCP) and photocurrent (OCC) measurements were executed in the following manner: 20 s dark, 40 s illumination (excitation), and 60 s dark (decay). During all photo-electrochemical experiments, the light source was a 300 W Xe arc lamp of ScienceTech’s Tunable Light Source, and the used irradiation was characterized by 100 mW cm^–2^ power density and spectral range of 300–1800 nm. Additionally, a filter was set—a quartz cuvette filled with distilled water to cut the infrared radiation.

## 3 Results and Discussion

**Figure 1:**
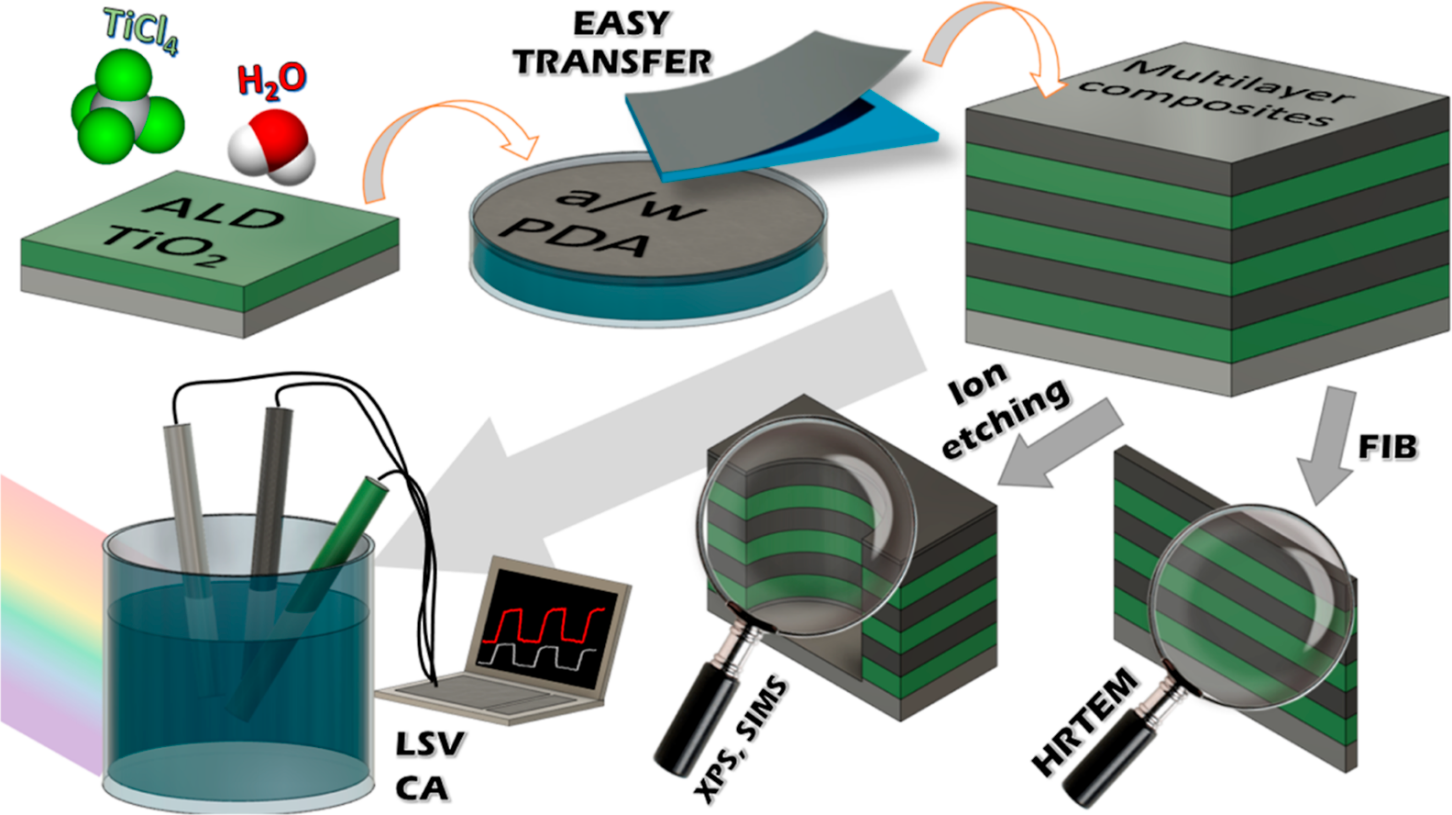
Scheme of the workflow–synthesis of the multilayer composites through ALD of the TiO_2_ layers and transfer of the PDA film from the air/water interface alternately until three-layer composites are obtained. Then structural, chemical, and electrochemical tests were carried on.

Our first goal was to obtain multilayer nanocomposites. Another goal was to generate a variable (gradient) nitrogen content in the TiO_2_ layers deposited on the PDA surfaces. To the best of our knowledge, such a method of doping TiO_2_ with nitrogen has not yet been described. Therefore, we conducted a series of experiments to investigate this phenomenon. First, Raman spectroscopy and XRD provided valuable information on the structural arrangement of PDA films obtained at the air/water interface and transferred ex situ to semiconductor surfaces. Then, cross sections (lamellas) of the samples were cut using the FIB. HRTEM showed the quality of the obtained interfaces and the thickness of individual layers. SIMS provided information on the element content in individual layers, allowing us to determine the gradient nature of doping and the layer interfaces. XPS was performed in the depth profiling mode, meaning that the etching of the sample surface preceded the spectrum’s collection to reach deeper layers. In this way, we thoroughly examined the chemical nature of the interface between PDA and nitrogen-doped TiO_2_ layers. Optical tests using UV–vis spectroscopy and ellipsometry showed a reduction in the band gap with each subsequent layer and a change in the optical properties of TiO_2_ due to nitrogen doping. Finally, LSC and CA showed multilayer composites’ more significant application potential than TiO_2_ and single-layer composites. Schematically, the course of the experiment is presented in Figure 1.

**Figure 2:**
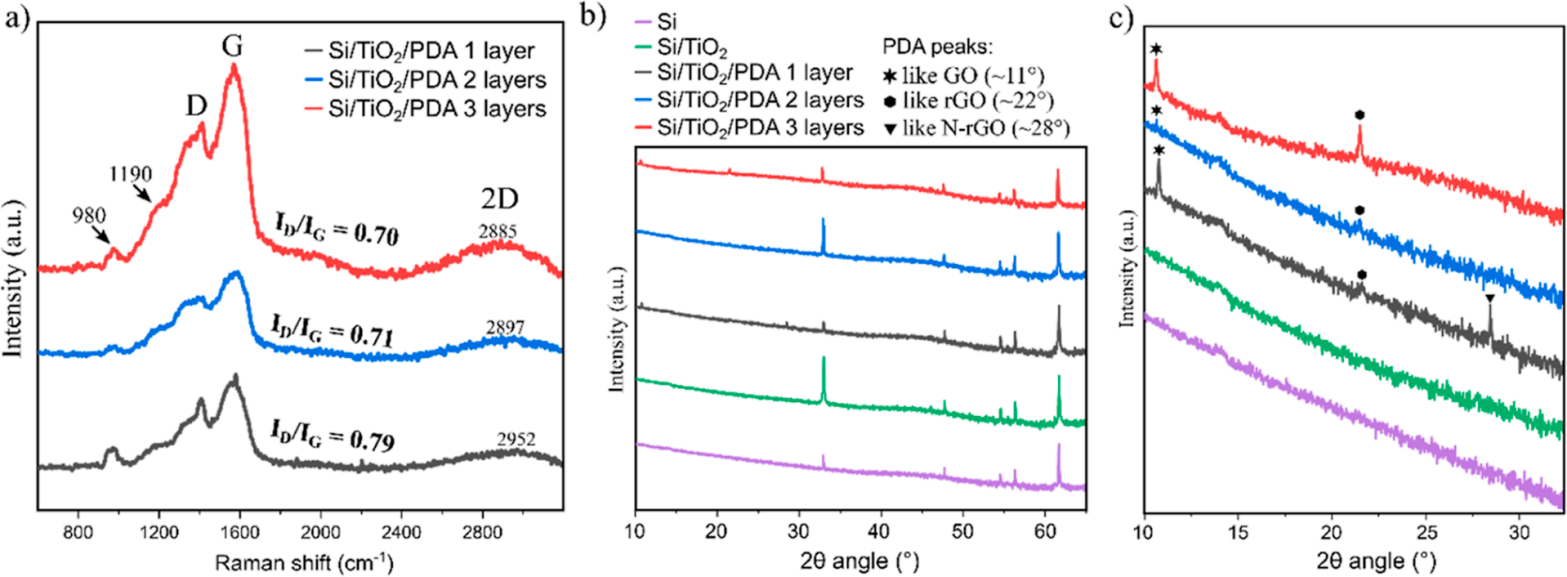
Raman spectra of the multilayer TiO_2_/PDA nanocomposites (a) and X-ray diffractogram of the Si substrate, Si/TiO_2_, and multilayer composites in the 2θ range from 10 to 65° (b) and 10 to 35° (c).

Starting from Raman spectroscopy (Figure 2a), the band around 1190 cm^–1^ indicates an important PDA structural unit; it is NH in-plane deformation mode originating from the pyrrole rings.^16^ As we presented earlier,^14,15^ the occurrence of 2D, D, and G bands is typical for this type of PDA films transferred ex situ from the air/water interface. Interestingly, the increasing number of layers showed no significant shift of the D and G peaks. Their centers are around 1380 cm^–1^ (D) and 1570 cm^–1^ (G). In turn, the *I*_D_/*I*_G_ intensity ratio, widely used to evaluate the defect density in graphene or graphite-like carbon-based materials,^40^ varies. The comparison demonstrates the decreasing defect density with increasing number of layers. It has previously been described that high temperature^40^ or laser annealing^41,42^ of the PDA may reduce defect density. However, this result was unexpected since the low temperature used in the ALD process (for deposition of TiO_2_ layers) should not lead to PDA graphitization. Notwithstanding, a dehydration reaction occurring normally in this temperature range in the PDA layer would reduce the concentration of hydroxyl groups present at the PDA surface,^43^ thus converting the more GO-like structure into a more rGO-like one. Moreover, the 2D peak is shifting toward lower wavenumbers with increasing layers, further suggesting transformation into rGO-like structure.^44^

The XRD patterns are dominated by peaks originating from the Si wafer used as the substrate. In turn, the lack of clear peaks that could be assigned to crystalline TiO_2_ confirms the amorphous nature of these layers (Figure 2b). Therefore, a close-up of the 2θ angle section from 10 to 30° was made and shown in Figure 2c to study a region of peaks typical for materials such as GO and rGO. The one-, two- and three-layer composites show GO-like and rGO-like peaks, but the latter is most significant in the case of the three-layer composite, which confirms the previous observations based on Raman spectroscopy. However, attention should be paid to the lack of a peak typical for N-rGO,^45,46^ i.e., nitrogen-doped reduced graphene oxide, in the case of two- and three-layer composites. Once again, the relatively low temperature and generally mild conditions of the TiO_2_ ALD process should not lead to the decomposition of the N-rGO.^47^ Instead, we postulate that a new N-TiO_2_ species may be formed at the PDA/TiO_2_ interface, as further results show.

**Figure 3:**
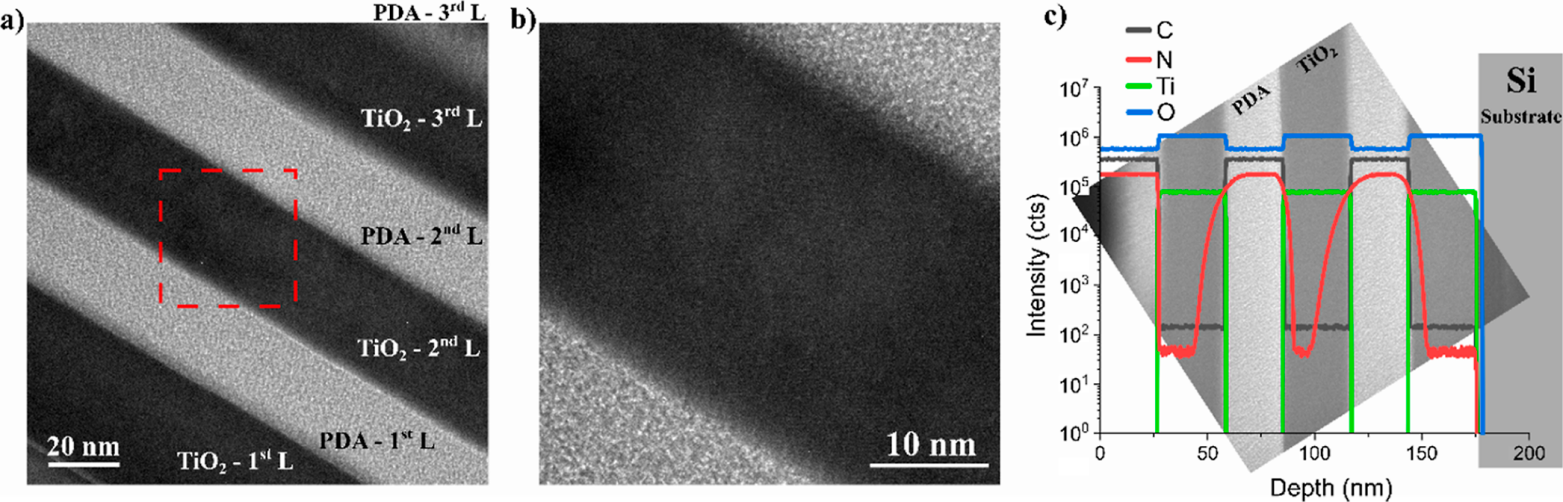
HRTEM image of the cross-section of the TiO_2_/PDA three-layer nanocomposite (a), close-up of the interface regions (b), and SIMS depth profile of the TiO_2_/PDA three-layer nanocomposite together with the HRTEM image of the cross-section in the background for better illustration of the multilayer structure (c).

A HRTEM image of the three-layer composite cross-section is shown in Figure 3a, and a close-up is shown in Figure 3b. Unfortunately, the top layer of the PDA was significantly damaged during the FIB cutting process (see the Materials and Methods section); therefore, it is not presented in the image. However, six extremely sharp and perfectly defined layers (Si/TiO_2_/PDA/TiO_2_/PDA/TiO_2_) can be seen; their thickness is approximately 25 nm (PDA) and 40 nm (TiO_2_). The structure of the obtained laminar composite consists of alternately arranged thin, large-surface layers separated by clear sharp boundaries with no irregularities. It would be impossible to obtain such structures using the in situ PDA deposition method because the layers deposited in this way are amorphous and morphologically irregular.

Figure 3c shows the obtained SIMS depth profile of the three-layer composite sample, superimposed on the HRTEM image to better visualize the element content in individual layers. For clarity and better visualization, the depth profile starts from the top PDA layer, i.e., the one deposited last on the composite surface. Going deeper, we reach subsequent layers. The second and third TiO_2_ layers (in the order in which they were obtained) were deposited on the PDA surface. The titanium spectrum can be used as a benchmark to precisely determine the interfaces between PDA and TiO_2_. It is also visible that the TiO_2_ layer has a higher oxygen signal intensity than PDA, further confirming sharp boundaries. The carbon signal in the TiO_2_ layers is very low and is related to the presence of residual contamination, but its concentration was estimated to be in the part-per-million range. In the case of mixing of phases or carbon doping, we would note a gradient of its content in the TiO_2_ layer, which we do not observe. On the other hand, a clear gradient of nitrogen content can be seen in the TiO_2_ layer’s growth on the PDA surfaces, starting from high content at the interface, decreasing gradually deep into the TiO_2_ layer, until rapidly increasing close to the next interface with PDA deposited onto the TiO_2_ surface. We postulate nitrogen migration from PDA during the TiO_2_ growth process using the ALD method, where PDA acts as a polymeric nitrogen source. As a reminder, the conditions of the ALD process were as follows: temperature 200 °C, time about 12 h, and the atmosphere was a vacuum alternating with argon (for a more detailed description, see the Materials and Methods section). In the later paragraphs, we show further evidence of TiO_2_ N doping via ALD with polymer nitrogen sourcing.

**Figure 4:**
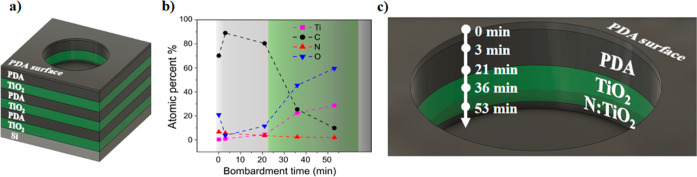
Scheme of the TiO_2_/PDA three-layer nanocomposite structure and etching depth (a), XPS in-depth profile of the atomic percentage in the two top layers (b), and visualization of the etching depth (c).

We used the XPS in-depth profile mode for two purposes. First, we examine the elemental content in the two top layers of the three layer sample. This helped us determine the speed of the Ar^+^ ion etching process and the location where each measurement was made (Figure 4). Figure 4a schematically shows the multilayer composite with etching depth, which will be helpful when discussing the XPS spectroscopy results.

Five spectra were analyzed, for which the etching time was 0, 3, 21, 36, and 53 min. Figure 4b shows the atomic elemental content of each spot reached after a given etching time. It was possible to deduce the penetration depth using this information and the previously described results (HRTEM and SIMS). Therefore, for an etching time of 0 min, the analyzed spot is on the surfaces of the top PDA layer; for 3 min, the bulk PDA layer; for 21 min, the PDA/TiO_2_ interface; for 36 min, bulk TiO_2_ (low N content); and finally, for 53 min, bulk N-TiO_2_ with high nitrogen content. It is schematically shown in Figure 4c.

**Figure 5:**
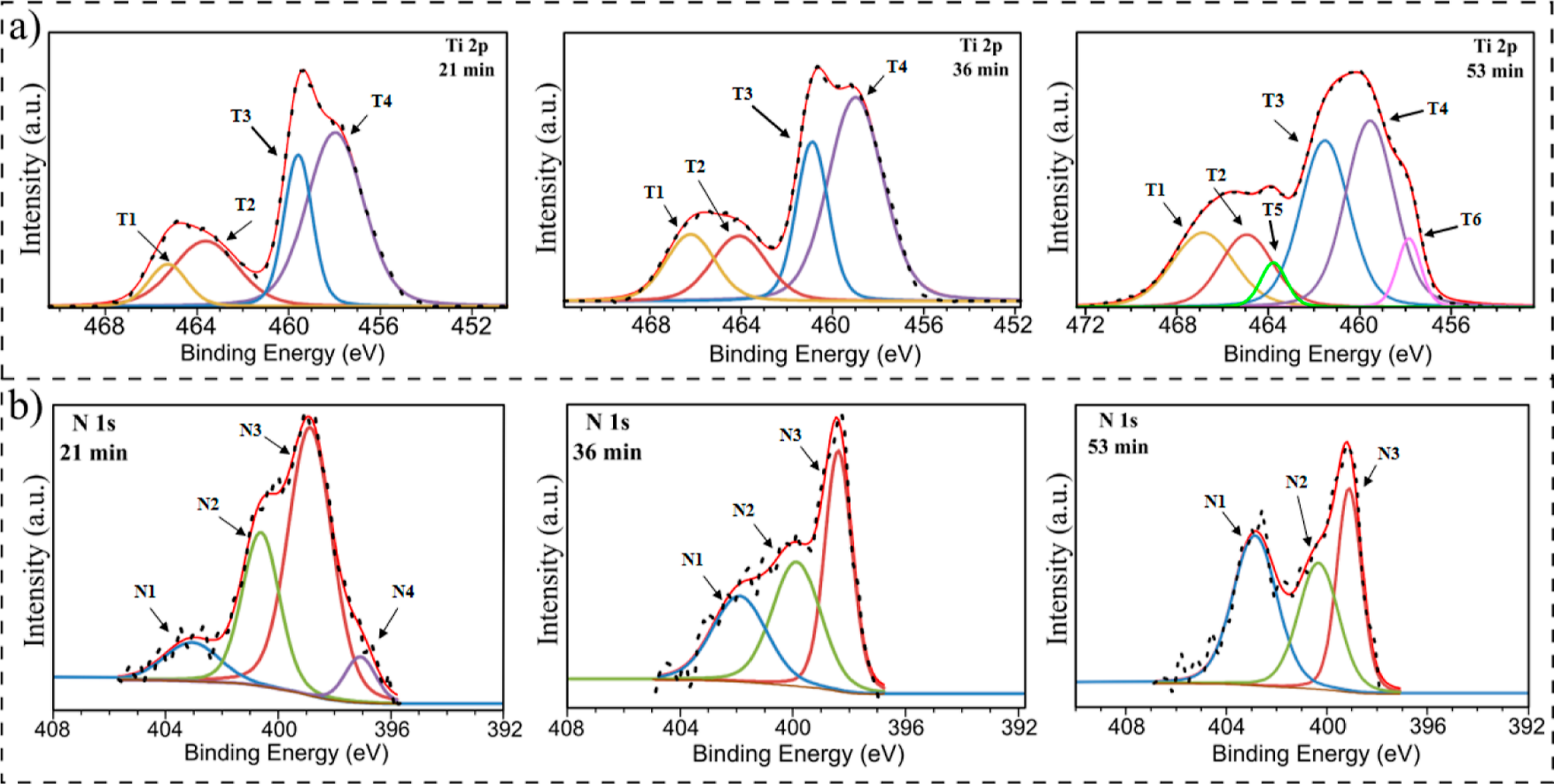
XPS high-resolution spectra of the Ti 2p (a) and N 1s (b) regions for etching times 21, 36, and 53 min.

Then, with this knowledge, we analyzed the high-resolution spectra of the key regions (Figure 5). However, bearing in mind the main drawbacks of the ion-etching method, it is preferential sputtering (e.g., oxygen in metal oxides), a mixture of nonuniform sputtering (e.g., cratering) and chemical reduction due to sputtering,^48^ we mainly focus on the analysis of Ti 2p (Figure 5a) and N 1s (Figure 5b) regions. Oxygen and carbon from the first PDA layer undergo many of the effects mentioned above (etching of polymers results in their chemical degradation^49^), contaminating the TiO_2_ layer and affecting the measurement. However, Supporting Information includes full XPS spectra for all samples and high-resolution spectra of the O 1s and C 1s regions (Figures S1–S5). In turn, the interactions between nitrogen and titanium should be exclusively caused by forming some new species at the interface and, therefore, nitrogen migration into the TiO_2_ layers. The information about peaks in the Ti 2p and N 1s regions is also summarized in Table 1 below.

**Table 1:** XPS Depth Profiles—Area Percentage, Binding Energies, and FWHM Values for Ti 2p and N 1s Regions for Etching Times 21, 36, and 53 min

etching time	peak	binding energy (eV)	fwhm	area %
21 min	T1	465.27	1.95	8.0
	T2	463.63	3.32	21.1
	T3	459.58	1.46	21.5
	T4	457.97	2.92	49.4
	N1	403.06	2.31	10.1
	N2	400.62	1.55	27.0
	N3	398.86	1.85	56.2
	N4	397.08	1.28	6.7
37 min	T1	466.22	2.54	14.2
	T2	464.10	2.84	15.5
	T3	460.89	1.65	22.0
	T4	458.99	2.83	48.3
	N1	401.89	2.28	27.0
	N2	399.87	2.03	34.9
	N3	398.39	1.14	38.1
53 min	T1	466.85	3.20	16.2
	T2	464.94	2.72	13.4
	T3	463.77	1.34	4.0
	T4	461.52	2.52	28.5
	T5	459.55	2.53	32.1
	T6	457.85	1.23	5.7
	N1	402.86	2.07	39.5
	N2	400.33	1.89	30.4
	N3	399.11	1.16	30.1

Starting from fitting the Ti 2p high-resolution spectra, T1 and T3 peaks were assigned to 2p_1/2_ and 2p_3/2_ energy levels of Ti^4+^, respectively.^50−52^ Next, T2 and T4 were appointed to 2p_1/2_ and 2p_3/2_ energy levels of Ti^3+^, respectively.^50−52^ The latter suggests the formation of oxygen vacancies^53^ or TiN_*x*_ species.^54^ However, this can be partially or exclusively the effect of the Ar ion etching process.^55^ In turn, the T5 and T6 pair is originating most probably from titanium oxynitride.^56^ This observation is also supported by the decrease in the overall area of Ti^4+^ peaks, which indicates Ti–O–N formation by substituting transition metal ions.^57^ The N4 peak is present only for the spectra after 21 min of etching, as described earlier (Figure 4); in this region, the PDA/TiO_2_ interface is present. It most likely indicates the coordination of nitrogen to titanium atoms on the TiO_2_ surface.^58,59^ This is particularly reasonable because PDA adheres to surfaces via catechol and amino groups containing nitrogen. The peaks N2 and N3 cannot be assigned with certainty because they overlap with peaks typical for PDA (primary and secondary amine groups),^16^ which are also visible for shorter etching times (Figures S1 and S2). However, it is visible that the intensity of N1 increases with an increasing etching time. According to the literature, it should be associated with N–Ti–O formation,^60^ and it is nitrogen doping via interstitial position occupation. Therefore, we showed that TiO_2_ is doped through both substitutive and interstitial nitrogen atoms and that the doping is gradient in nature (as previously shown by using SIMS). It is worth emphasizing that the multiple N doping types might induce the formation of new energy levels in the forbidden band of titanium oxide, resulting in shifting the absorption edge to lower photon energies and thus reducing the band gap of TiO_2_.

**Figure 6:**
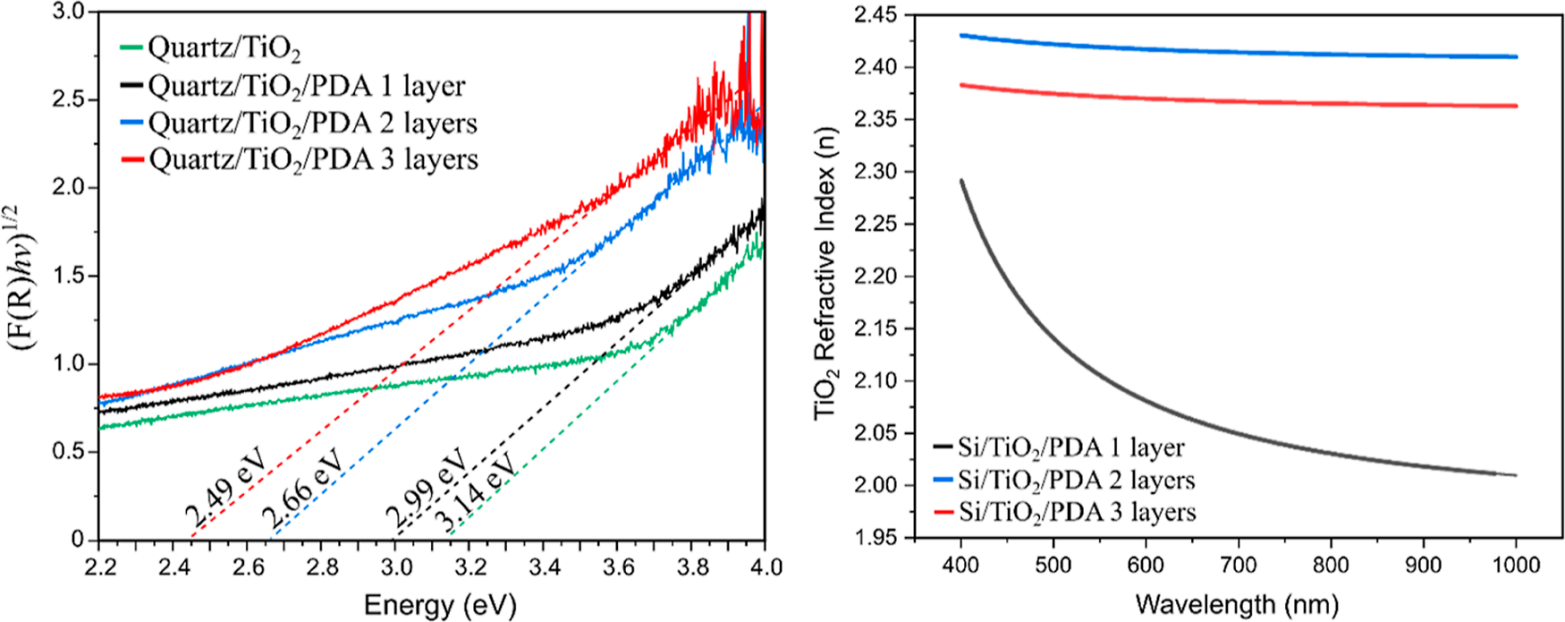
Tauc plot (UV–vis spectroscopy) of the quartz/TiO_2_ and quartz/TiO_2_/PDA multilayer composites (a) and the real part of the refractive index (*n*) vs wavelength graph of the TiO_2_ layers in Si/TiO_2_/PDA multilayer composites (b).

Therefore, the value of the band gap was examined. Figure 6a shows the Tauc plot for bare TiO_2_ and one-, two- and three-layer TiO_2_/PDA composites.

It is visible that with each layer the band gap value is lowered due to the phenomena described in the previous paragraph. The difference in the obtained value between subsequent samples is ∼0.15 eV, but the exception is the difference between one and two layers (0.33 eV). This is because for two layers, nitrogen doping already introduces new energy levels in the forbidden band of TiO_2_, unlike in the case of a one-layer composite where the only enhancement effect is the formation of the PDA/TiO_2_ interface.

To confirm these observations, we performed ellipsometric tests. The refractive index (*n*) describes the speed of light propagation through the material and strongly correlates with the electrical properties. Generally, the bandgap energy and refractive index values are inversely proportional for semiconductors.^61^ Moreover, it was previously shown that doping of titanium oxide can increase its refractive index.^62,63^ Our model calculated the refractive index for the TiO_2_ layers in each nanocomposite. For the record, two- and three-layer composites are characterized by gradient nitrogen doping of the TiO_2_ layer. This is visible in Figure 6b because the *n* value is higher within the entire investigated spectrum (from 400 to 1000 nm). This further confirms the postulated doping mechanism and the reduction of the bandgap energy. We also determined the real part of the refractive index (Figure S6a) and extinction coefficient (Figure S6b) for PDA layers. According to our knowledge, these values were determined for the first time for this type of thin PDA film from the air/water interface, which may constitute essential reference data for other experiments. The extinction coefficient has not changed significantly, but the real part of the refractive index of PDA increases with the increasing number of layers. This is related to the XRD results, which showed that PDA in three-layer composites shows structural features more similar to rGO than in the case of one-layer and two-layer composites. It was previously shown that rGO coatings have a higher *n* value than GO.^64^

**Figure 7:**
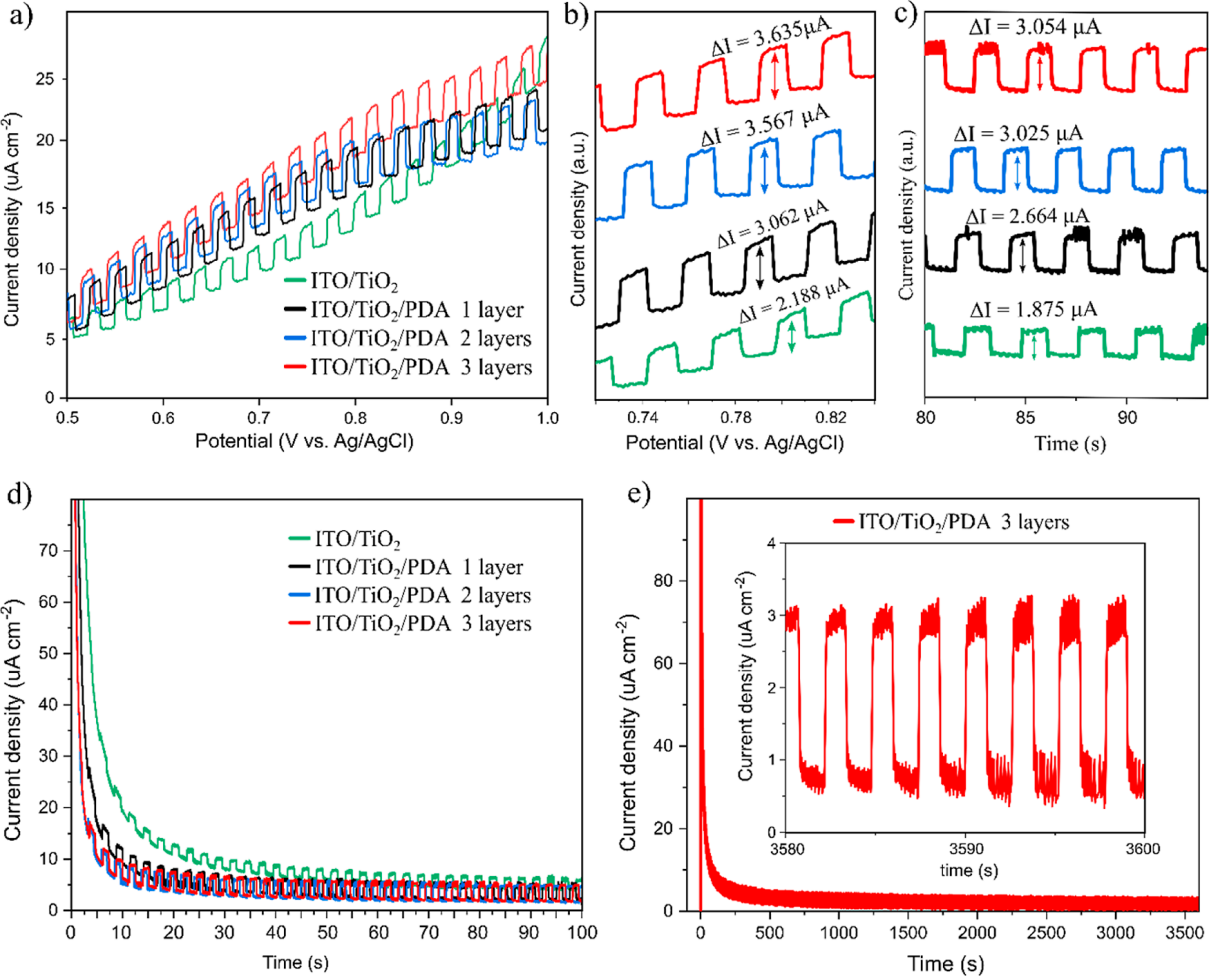
LSV under chopped illumination (a), close-up of the ∼0.8 V region (b), close-up of the 80–100 s region of the CA under chopped illumination (c), CA equilibrium during 100 s (d), and stability during 1 h (e).

The band gap reduction should positively affect the photochemical properties of the obtained composites, which is why we conducted LSV under chopped light illumination and CA under chopped light illumination tests (Figure 7).

LSV was performed in the positive potential region (0.5–1 V) under chopped UV–vis light illumination (Figure 7a) to measure the photogenerated current increase. A significant change was observed, as shown in the close-up of the ∼0.8 V region (Figure 7b). The percentage increase in photogenerated current was calculated and is presented in Table 1; as observed, there is a particularly big difference between TiO_2_ vs TiO_2_/PDA and between TiO_2_/PDA one layer vs TiO_2_/PDA two layers. As we described previously, this is related to forming a functional TiO_2_/PDA interface and the nitrogen doping of the TiO_2_ layer. The highest photocurrent was obtained for a three-layer composite.

The same phenomena were confirmed via the CA scan (Figure 7c). After reaching the equilibrium at the constant potential set on 1 V, the photocurrent induced again by chopped light illumination was measured. Again, there was a significant increase in the photocurrent, as presented in Table 2. The results obtained with the LSV and CA methods are very similar. A more detailed discussion of the CA results is provided in the next paragraph. The LSV photocurrent does not exhibit a significant increase as the applied potential becomes more positive. This is probably due to the lack of an efficient electron transport layer in the system and provides a perspective for future research.

**Table 2:** Photogenerated Currents of the Multilayer Composites and Increases in Their Value Compared to Bare TiO_2_^a^

method	LSV (at ∼0.8 V)			CA (at ∼80 s, 1 V)		
sample	1L	2L	3L	1L	2L	3L
photocurrent (μA)	3.062	3.567	3.635	2.664	3.025	3.054
photocurrent increase vs TiO_2_ (%)	39.95	63.03	66.13	42.08	61.33	62.88

a 1L, 2L, 3L—PDA/TiO_2_ one layer, two layer, and three layer composites, respectively.

In CA, the current was recorded under chopped UV–vis illumination for 100 s while the sample approached an equilibrium state after increasing the potential from 0 to 1 V (Figure 7d). After the initial rapid current increase, the current density drops exponentially due to the electrical barrier at the solid/electrolyte interface. The switching time was determined as the time for a system to reach 90% of its full current density drop. Calculated times were 0.42, 0.27, 0.14, and 0.12 s for TiO_2_, one layer TiO_2_/PDA, two layers, and three layers, respectively. In the course of previous tests, the three-layer composite sample showed the most outstanding performance (smallest band gap, largest photocurrent, and shortest switching time). Therefore, we decided to test its stability (Figure 7e) by applying a constant potential (1 V) while maintaining chopped illumination for 1 h (3600 s). After an initial exponential decay, the current density is stabilized at values around 1 μA cm^–2^ (light off) and 3 μA cm^–2^ (light on) and remains at this level throughout the experiment, as shown in the inset graph. This indicates the excellent stability of the multilayer composites. Lastly, we investigated the OCP and OCC of the three-layer nanocomposite, to provide data for future experiments or comparative analyses. For clarity, OCP and OCC under the dark conditions have been subtracted. In Figure S7, the typical behavior of a photocatalyst can be observed, i.e., rapid step after illumination and a noticeably slower decay after turning off the light, depending on photogenerated charge carriers’ lifetimes. The maximum OCP immediately after illumination was −55 mV, while the maximum OCC was 7 nA.

## 4 Conclusions

Overall, this study introduced an innovative approach to nitrogen doping in amorphous TiO_2_ thin films and the production of polymer/oxide nanocomposites tailored for photocatalytic applications. Central to this advancement is the utilization of a unique method for creating PDA free-standing films at the air/water interface combined with ALD of the oxide layer. This technique enhances the efficacy of the nitrogen doping process and significantly improves the structural and functional qualities of the resultant polymer/oxide nanocomposites, making them more suitable for a range of uses. We obtained TiO_2_ layers with gradient nitrogen doping by ALD with polymer nitrogen sourcing at low temperature (200 °C). This approach allowed us to preserve the amorphous structure of TiO_2_ (which crystallizes at higher temperatures) and the 2D-like layered structure of the PDA films from the air/water interface, which undergoes carbonization at higher temperatures. We showed with XPS depth profiling that TiO_2_ is doped through both substitutive and interstitial nitrogen atoms, which is remarkably important from the point of view of applications in photocatalysis because it provides new energy levels inside the forbidden band and thus lowers the semiconductor band gap, allowing for a larger range of light absorption. Additionally, there is a synergistic effect of the previously mentioned phenomenon and PDA/TiO_2_ interfaces, which are well preserved in our method, as evidenced by sharp interfaces visible in the HRTEM images and SIMS profiles. The mere presence of the PDA/TiO_2_ interface contributed to reducing the bandgap energy by 0.15 eV (for the one-layer sample), while obtaining multilayer composites with nitrogen-doped titanium oxide layers resulted in a further reduction of this value by another 0.33 and 0.15 eV for the second and third layers, respectively. The total reduction for the three-layer composite compared to TiO_2_ was 0.63 eV.

Furthermore, we showed that our layered nanocomposites are characterized by greater photo-electrochemical activity because in both the case of LSV and CA under chopped light illumination, the photogenerated current was increased by more than 60% for two- and three-layer composites in comparison to bare TiO_2_. The stability of these materials was also proven as there was no significant reduction in the photocurrent value during the 1 h CA experiment. At the same time, we perceive much room for further improvement of the photocatalytic properties of the materials we presented. We anticipate that the critical development direction is to enrich the composite with an electron transport layer and a surface catalyst (e.g., nanoparticle decoration) to increase the photo-electrochemical activity. However, the simple, cheap, and effective method we have presented for obtaining gradient nitrogen-doped TiO_2_ opens new perspectives for nanomaterials based on polymer/inorganic semiconductor heterojunctions, especially those where it is required to significantly reduce the band gap without damaging the delicate interface structure.

Finally, further studies should address optimization and design aspects of the architecture here, such as the influence of layer thickness on the total N doping migration, deposition temperature, and other postannealing effects. We also anticipate that the methodology presented here could be exploited in other N-accepting photoactive oxides and ceramic materials.

## Data Availability

The raw/processed data required to reproduce these findings cannot be shared at this time as the data also forms part of an ongoing study. Data might be shared upon reasonable request to the corresponding author.

## Supporting Information Available

XPS atomic percentage and binding energies of the elements for etching times 0, 3, 21, 36, and 53 min; XPS area percentage, fwhm, and binding energies of high-resolution regions for etching times 0, 3, 21, 36, and 53 min; XPS full spectra and high-resolution spectra for etching times 0, 3, 21, 36, and 53 min; real part of the refractive index and the extinction coefficient for the PDA; and OCP and OCC curves for the three-layer nanocomposite (PDF)
