# Supplementary material for: TiO2/PDA Multilayer Nanocomposites with Exceptionally Sharp Large-Scale Interfaces and Nitrogen Doping Gradient
Source: ACS Appl Mater Interfaces. 2024 Feb 13;16(8):10774–84. doi: 10.1021/acsami.3c18935 (PMC10910457; doi:10.1021/acsami.3c18935)
Supplement: Supplementary file 1 — am3c18935_si_001.pdf [file am3c18935_si_001.pdf]

Supporting Information

TiO_2/polydopamine multilayer nanocomposite with exceptionally sharp large-scale interfaces and nitrogen doping gradient

Jakub Szewczyk*[1,2], Igor Iatsunskyi[1], Paweł Piotr Michałowski[3], Karol Załęski[1], Cassandre Lamboux[2], Syreina Sayegh[2], Elissa Makhoul[2], Andreu Cabot[4,5], Xingqi Chang[4], Mikhael Bechelany[2,6], Emerson Coy[1]*

[1] NanoBioMedical Centre, Adam Mickiewicz University, Wszechnicy Piastowskiej 3, 61-614, Poznan, Poland.
[2] Institut Européen des Membranes, IEM, UMR 5635, Univ Montpellier, CNRS, ENSCM Place Eugène Bataillon, 34095 Montpellier cedex 5, France
[3] Łukasiewicz Research Network - Institute of Microelectronics and Photonics, Aleja Lotników 32/46, 02-668 Warsaw, Poland
[4] Advanced Materials Department, Catalonia Institute for Energy Research (IREC), Sant Adrià de Besòs, 08930, Barcelona, Spain
[5] ICREA, Pg. Lluís Companys 23, 08010 Barcelona, Spain
[6] Gulf University for Science and Technology, GUST, Kuwait

*** Corresponding authors:** jaksze3@amu.edu.pl, coyeme@amu.edu.pl

Table of contents

**Table S1**	XPS Atomic percentage and binding energies of the elements for etching times 0, 3, 21, 36 and 53 min.	S2
**Table S2**	XPS Area percentage, FWHM and binding energies of high resolution regions for etching times 0, 3, 21, 36 and 53 min.	S3
**Figure S1**	XPS Full spectra and High-Resolution Spectra for etching time 0 min	S4
**Figure S2**	XPS Full spectra and High-Resolution Spectra for etching time 3 min	S5
**Figure S3**	XPS Full spectra and High-Resolution Spectra for etching time 21 min	S6
**Figure S4**	XPS Full spectra and High-Resolution Spectra for etching time 36 min	S7
**Figure S5**	XPS Full spectra and High-Resolution Spectra for etching time 53 min	S8
**Figure S6**	The real part of the refractive index and the extinction coefficient for the PDA	S9
**Figure S7**	Open Circuit Photopotential (a), Open Circuit Photocurrent (b) curves for 3-layer nanocomposite.	S10

Supporting Information

Table S1. X-Ray photoelectron spectroscopy deep profiles, Atomic percentage and binding energies of the elements for etching times 0, 3, 21, 36 and 53 min.

Etching time (min)	Element	Binding Energy (eV)	Atomic %
0	Ti	458.83	0.3
	C	284.83	70.1
	N	400.13	6.6
	O	532.98	21.0
	Si	101.93	1.0
3	Ti	459.41	0.9
	C	284.71	89.0
	N	399.21	5.6
	O	531.11	3.8
	Si	-	-
21	Ti	459.28	4.5
	C	284.73	80.4
	N	398.98	3.6
	O	531.18	11.6
	Si	-	-
37	Ti	460.67	22.6
	C	286.07	25.5
	N	398.27	2.4
	O	532.42	45.5
	Si	103.77	2.6
53	Ti	460.09	28.7
	C	286.89	9.9
	N	399.19	1.9
	O	533.09	59.6
	Si	-	-

Table S2. X-ray photoelectron spectroscopy deep profiles- area percentage, binding energies and FWHM values for C 1s, O 1s regions for etching times 0, 3, 21, 36 and 53 min. Additionally, the N 1s region for times 0 and 3 min was shown (not shown in Table 1)

Etching time (min)	High resolution region	Binding Energy (eV)	FWHM	Area %
0	C 1s	290.19	3.85	2.7
		288.31	1.81	7.0
		286.18	1.64	43.9
		285.21	0.76	7.3
		284.68	1.08	39.1
	N 1s	401.81	2.32	25.5
		400.09	1.85	73.0
		398.67	0.79	1.53
	O 1s	533.02	1.78	69.8
		531.49	1.88	30.2
3	C 1s	289.88	1.71	0.7
		287.95	2.49	4.5
		285.79	2.16	26.8
		285.10	1.47	40.1
		284.59	1.01	28.0
	N 1s	402.31	1.86	4.8
		400.38	1.57	33.2
		398.95	1.71	62.1
	O 1s	532.66	2.44	50.6
		531.04	1.34	49.4
21	C 1s	290.59	2.07	1.6
		288.51	2.25	4.1
		286.25	2.49	20.6
		285.16	1.67	44.8
		284.63	1.02	29.0
	O 1s	532.50	2.49	39.0
		531.15	1.38	61.0
36	C 1s	288.72	1.87	3.5
		287.22	1.62	18.5
		286.03	1.42	64.8
		284.62	1.25	13.1
	O 1s	533.19	1.89	37.7
		532.32	1.19	62.3
53	C 1s	288.33	1.77	13.4
		286.90	1.63	61.0
		284.80	1.16	25.6
	O 1s	534.06	2.08	36.8
		533.09	1.23	63.2

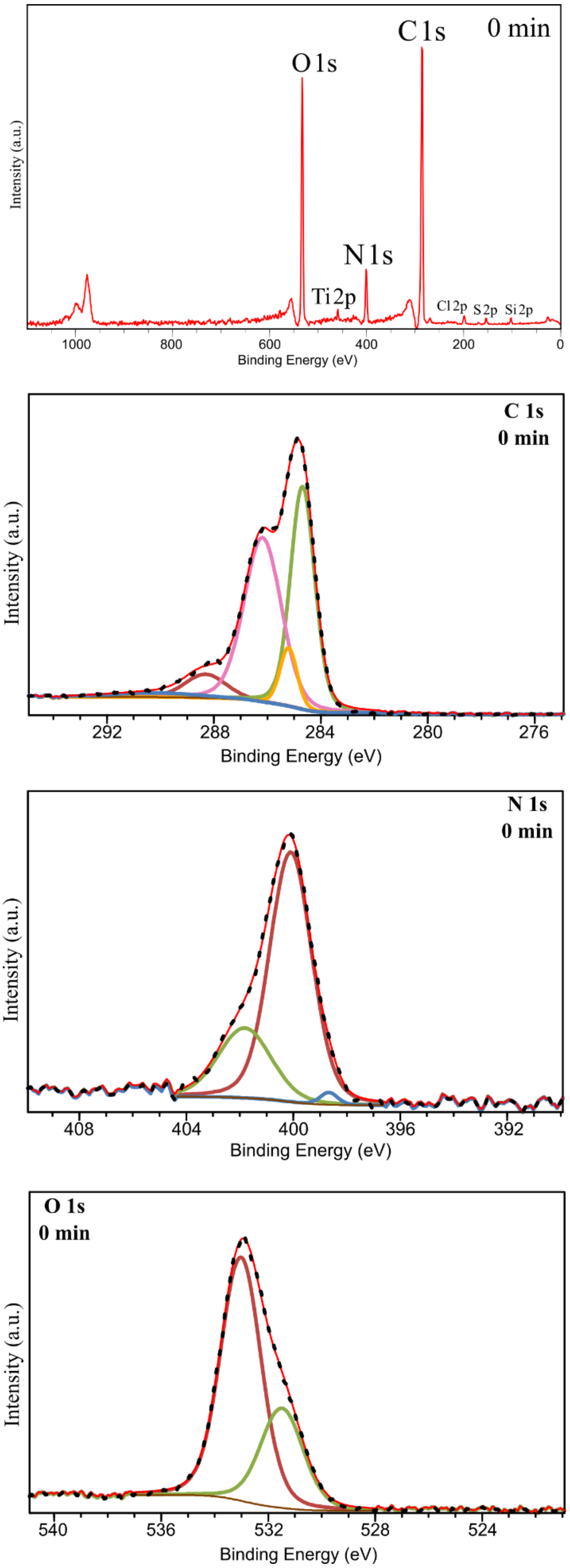

Figure S1. X-ray Photoelectron Full spectra and High-Resolution Spectra of the C 1s, N 1s and O 1s regions for etching time 0 min.

Supporting Information

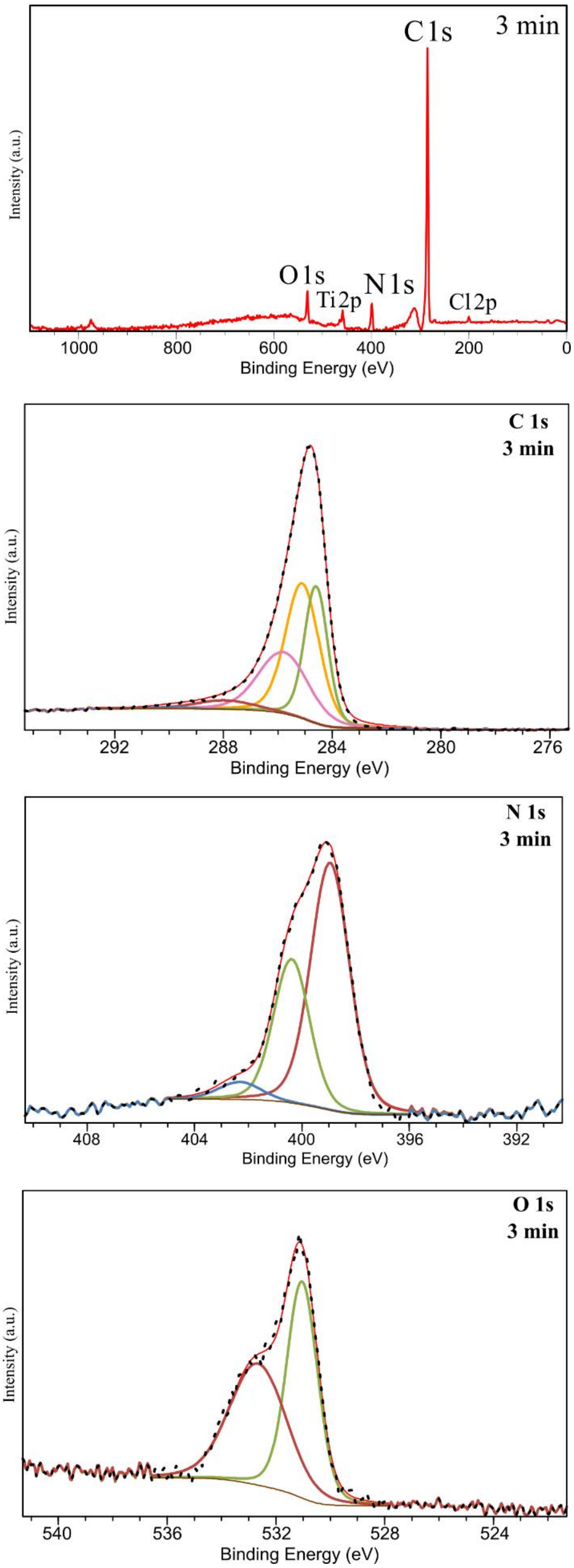

Figure S2. X-ray Photoelectron Full spectra and High-Resolution Spectra of the C 1s, N 1s and O 1s regions for etching time 3 min.

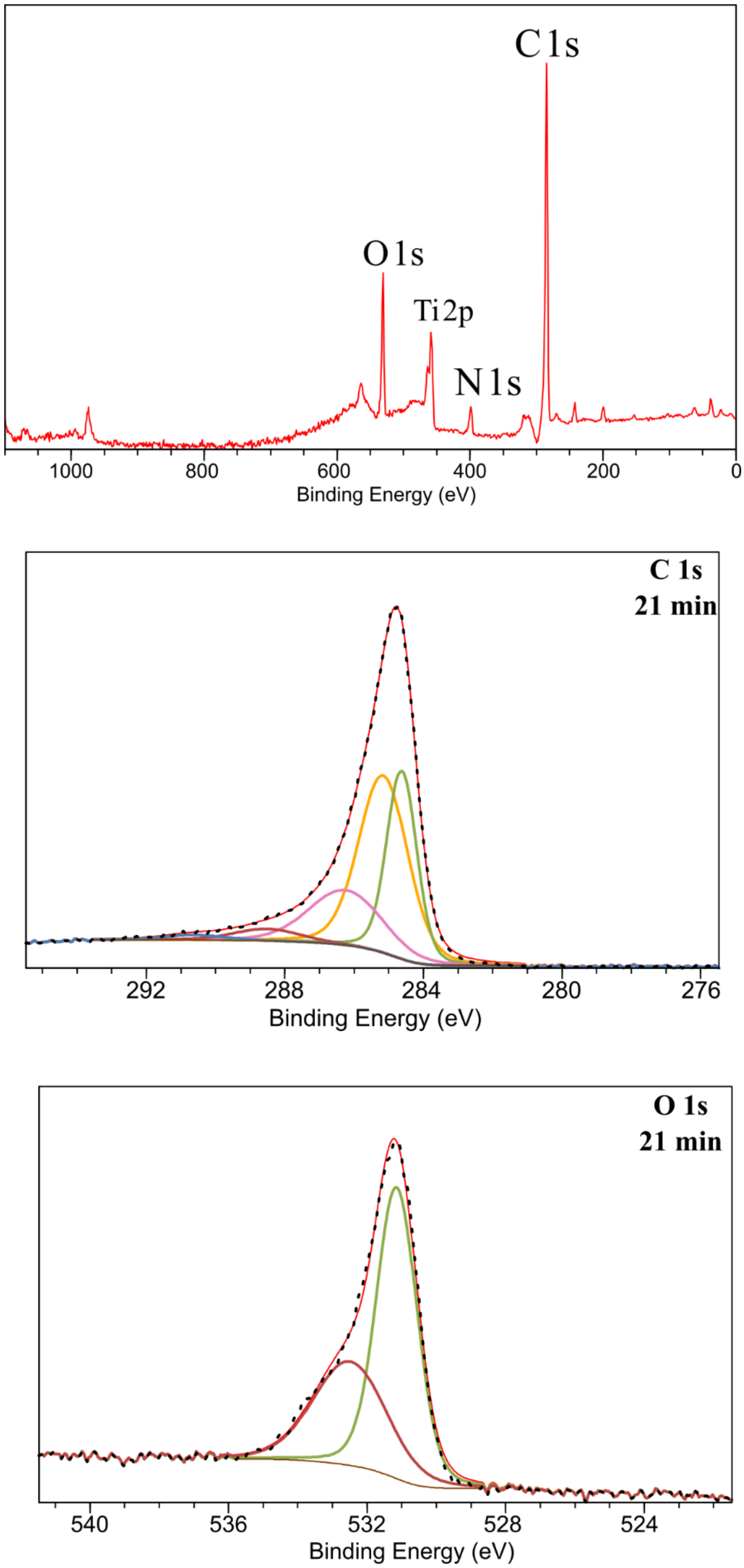

Figure S3. X-ray Photoelectron Full spectra and High-Resolution Spectra of the C 1s and O 1s regions for etching time 21 min.

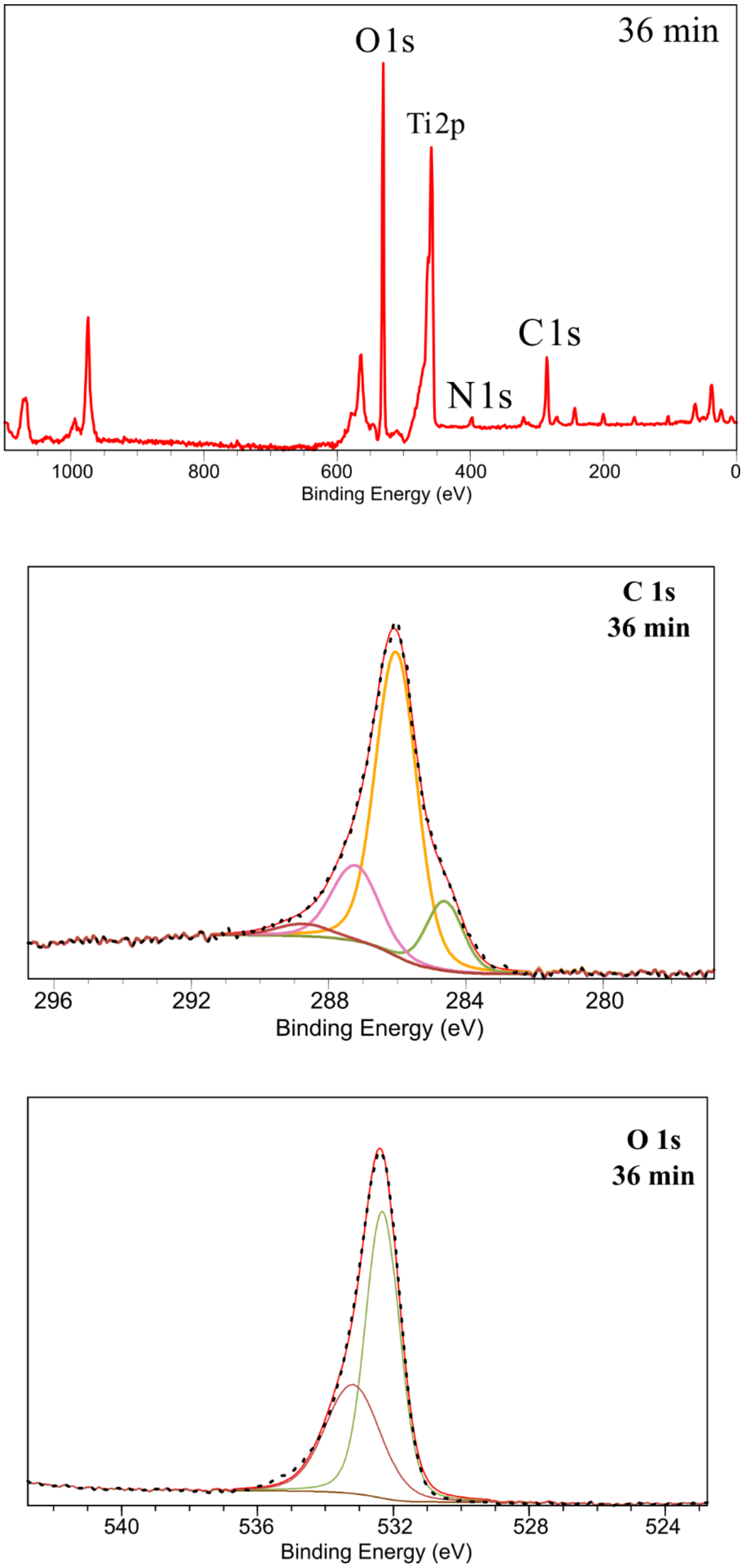

Figure S4. X-ray Photoelectron Full spectra and High-Resolution Spectra of the C 1s and O 1s regions for etching time 36 min.

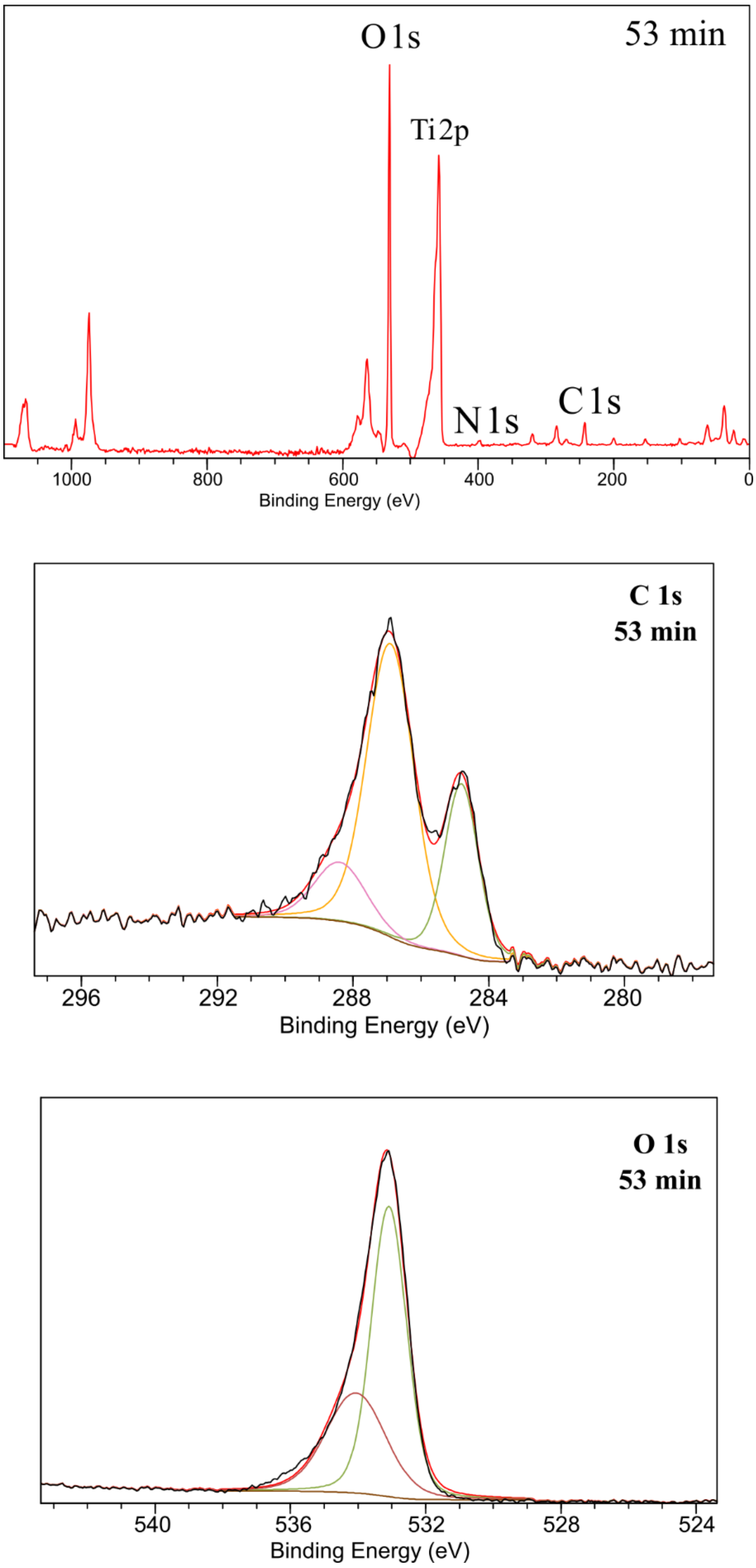

Figure S5. X-ray Photoelectron Full spectra and High-Resolution Spectra of the C 1s and O 1s regions for etching time 53 min.

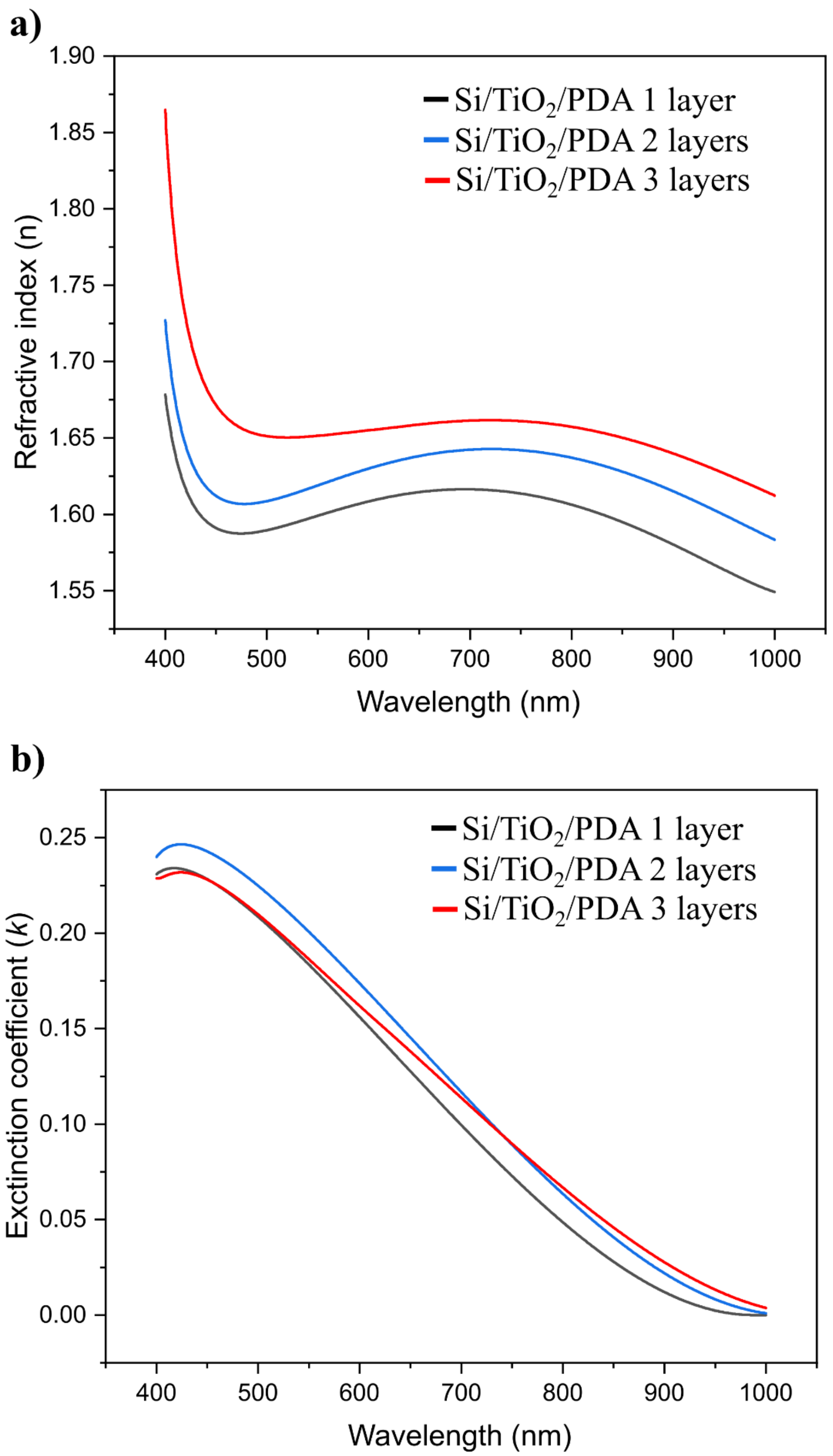

Figure S6. The real part of the refractive index (a), the extinction coefficient (b) for the PDA layers measured with ellipsometry.

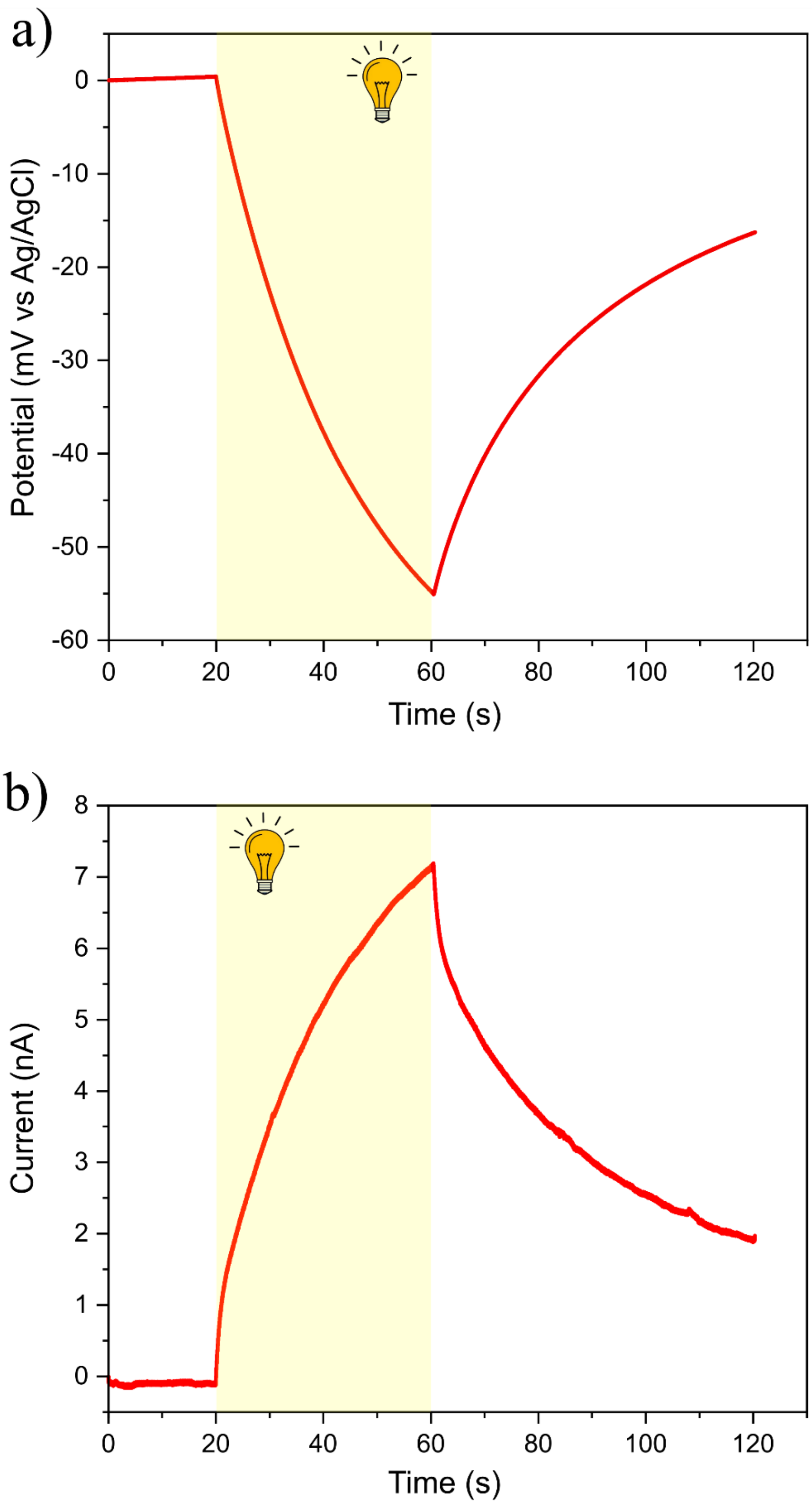

Figure S7. Open Circuit Photopotential (a), Open Circuit Photocurrent (b) curves for 3-layer nanocomposite.
